# Recent studies on betulinic acid and its biological and pharmacological activity

**DOI:** 10.17179/excli2015-150

**Published:** 2015-02-02

**Authors:** Sook Young Lee, Haeng Hoon Kim, Sang Un Park

**Affiliations:** 1 Regional Innovation Center for Dental Science & Engineering, Chosun University, 309 Pilmun-daero, Dong-gu, Gwangju, 501-759, Korea; 2 Department of Well-being Resources, Sunchon National University, 413 Jungangno, Suncheon, Jeollanam-do, 540-742, Korea; 3 Department of Crop Science, Chungnam National University, 99 Daehak-ro, Yuseong-gu, Daejeon, 305-764, Korea

## ⁯

Dear Editor,

Betulinic acid (3β-hydroxy-lup-20(29)-en-28-oic acid, BA), a pentacyclic lupane-type triterpene, is widely distributed in the plant kingdom (Mukherjee et al., 2006[20]; Fulda, 2008[8]). Johann Tobias Lowitz isolated the reduced form of BA from plants in 1788 and found that it was a prominent outer-bark constituent in white-barked birch trees (Bag and Dash, 2011[2]). BA has a wide range of biological and medicinal properties, including anti-human immunodeficiency virus (HIV), antibacterial, antimalarial, anti-inflammatory, anthelmintic, antinociceptive, anti-herpes simplex viruses-1 (HSV-1), immune-modulatory, antiangiogenic, and anticancer activity (Yogeeswari and Sriram, 2005[38]; Gheorgheosu et al, 2014[10]). Furthermore, the anti-tumor activity of BA can help overcome resistance by inducing apoptosis in a variety of human cancers. 

Semi-synthetic derivatives of natural plant products continue to play an important role in drug discovery and development (Pan et al., 2010[22]). To improve the potency of BA, many derivatives have been synthesized and evaluated for biological/medicinal applications (Jonnalagadda et al., 2013[13]; Csuk, 2014[5]). Because of its range of biological properties, BA has attracted much attention in recent years in the pharmaceutical industry. Here, we summarize key recent studies performed to evaluate the biological and pharmacological activities of BA and its derivatives (Table 1(Tab. 1)). (References in Table 1: Lingaraju et al., 2015[18]; Xu et al., 2014[35]; Kim et al., 2014[15]; Tiwari et al, 2014[29]; Xia et al., 2014[34]; Soica et al., 2014[27]; Sousa et al., 2014[28]; Jin et al., 2014[12]; Godugu et al., 2014[11]; Park et al., 2014[23]; Yi et al., 2014[37]; Castro et al., 2014[4]; Afzal et al., 2014[1]; Gao et al., 2014[9]; Zhao et al., 2013[39]; Ding et al., 2013[7]; Baratto et al., 2013[3]; Damle et al., 2013[6]; Li et al., 2013[17]; Wan et al., 2013[31]; Kaur and Arora, 2013[14]; Quan et al., 2013[25]; Reiner et al., 2013[26]; Qian et al., 2012[24]; Yang et al., 2012[36]; Wang et al., 2012[33]; Tzakos et al., 2012[30]; Liu and Luo, 2012[19]; Nader and Baraka, 2012[21]; Wan et al., 2012[32]; Kim et al., 2012[15]).

EXCLI Journal 2015;14:199-203 – ISSN 1611-2156
Received: January 17, 2015, accepted: January 27, 2015, published: February 02, 2015

| Key message | Reference |
|---|---|
| BA protects the lungs against inflammation and could be a potential modulator of inflammation in sepsis-induced acute lung injury. | Lingaraju et al., 2015 |
| BA induces HeLa cell apoptosis by triggering both the endoplasmic reticulum pathway and the reactive oxygen species (ROS)-mediated mitochondrial pathway. | Xu et al., 2014 |
| BA mediates the anti-estrogenic effects of *Proteus vulgaris;* this suggests its potential use as a therapeutic agent in estrogen-dependent tumors. | Kim et al., 2014 |
| BA dose-dependently inhibits proliferation and induces apoptosis in neuroblastoma and melanoma cell line apoptosis. | Tiwari et al, 2014 |
| BA reduces lactase dehydrogenase (LDH) and creatine kinase (CK) release, prevents cardiomyocyte apoptosis, and alleviates the extent of myocardial ischemia/reperfusion injury. | Xia et al., 2014 |
| Combination of BA with cyclodextrin treatment enhances anti-proliferative activity and aids in preventing *in vivo* tumor development. | Soica et al., 2014 |
| The betulin (BE) derivative BT06 and BA derivative AB13 may be promising alternatives leishmaniasis therapies, particularly in combination with miltefosine. | Sousa et al., 2014 |
| BA derived from *Vitis amurensis* root plays a novel role in melanogenesis. This finding has advanced our understanding of cosmetic therapy to reduce skin hyperpigmentation. | Jin et al., 2014 |
| Spray drying BA is a superior alternative formulation that significantly increases BA oral bioavailability and enhances anticancer efficacy. | Godugu et al., 2014 |
| BA may prevent bone loss in patients with bone metastases and cancer treatment-induced estrogen deficiency. | Park et al., 2014 |
| BA may exert hepatoprotective effects by increasing antioxidant capacity, through the improvement of the tissue redox system, maintenance of the antioxidant system, and decreased lipid peroxidation in the liver. | Yi et al., 2014 |
| BA regulates glycemia through classical insulin signaling by stimulating GLUT4 synthesis and translocation. Additionally, it does not cause hypercalcemia, which is highly significant from a drug-discovery perspective. | Castro et al., 2014 |
| BA has thyroid-enhancing potential by lowering thyroid-stimulating hormone levels and reducing thyroid tissue damage, thereby minimizing the symptoms of hypothyroidism when used anaphylactically in rats. | Afzal et al., 2014 |
| BA induces eryptosis/erythroptosis in human erythrocytes through $Ca^{2+}$ loading and membrane permeabilization. | Gao et al., 2014 |
| A novel mechanism for BA-mediated ATP-binding cassette transporter A1 (ABCA1) expression has been proposed, which may provide new methods to modulate vascular inflammation and atherosclerosis. | Zhao et al., 2013 |
| Topomer CoMFA studies on 37 BA and BE derivatives confirmed their in vitro anticancer activity (reported as IC50 values) in HT29 human colon cancer cells. | Ding et al., 2013 |
| Several novel 2,4-dinitrophenylhydrazone BA derivatives were prepared by chemical and biotransformation methods using fungi and carrot cells. Some compounds showed significant cytotoxicity and selectivity against tumor cell lines. | Baratto et al., 2013 |

EXCLI Journal 2015;14:199-203 – ISSN 1611-2156
Received: January 17, 2015, accepted: January 27, 2015, published: February 02, 2015

| Key message | Reference |
|---|---|
| Histopathological analyses of tumors revealed decreased angiogenesis, proliferation, and invasion in BA-treated animals. This is one of the first studies demonstrating the *in vivo* antitumor activity of BA in MCF-7 breast cancer tumors in nude mice. | Damle et al., 2013 |
| Lamin B1, which plays a crucial role in pancreatic cancer, was significantly downregulated by BA treatment in pancreatic cancer cells *in vitro* and in xenograft models. | Li et al., 2013 |
| BA or BE may ameliorate acute ethanol-induced fatty liver via toll-like receptor 4 (TLR4) and signal transducer and activator of transcription 3 (STAT3) *in vivo* and *in vitro*, suggesting it may be a promising agent for ethanol-induced fatty liver therapies. | Wan et al., 2013 |
| BA modulates the activity of xenobiotic and antioxidative enzymes with putative roles in cancer initiation and proliferation. | Kaur and Arora, 2013 |
| BA ameliorates intracellular lipid accumulation in liver cells, and may be a potential therapeutic for the prevention of fatty liver disease. | Quan et al., 2013 |
| BA inhibits deubiquitinases and induces apoptotic cell death in prostate cancer, but not normal, cells and tissues. Thus, it may be an effective, non-toxic, and clinically selective chemotherapeutic. | Reiner et al., 2013 |
| BA prevents endothelium-dependent relaxation (EDR) impairment in rat aortas exposed to exogenous superoxide anion, which may closely relate to oxidative stress reduction and endothelial nitric oxide synthase (eNOS)-nitric oxide (NO) pathway activation. | Qian et al., 2012 |
| BA induces apoptosis and blocks autophagic flux in KM3 cells. Both caspase 3 activation and autophagic flux inhibition contribute to BA-mediated apoptosis. | Yang et al., 2012 |
| BA may exert its anti-tumor effects by inducing tumor cell apoptosis. This process may also improve the immune response. | Wang et al., 2012 |
| BA may serve as an antithrombotic compound via antiplatelet activity. The antiplatelet effect of BA has been suggested to be similar to that prostacyclin (PGI2) receptor agonists; however, this requires further investigation. | Tzakos et al., 2012 |
| BA may be a potent and effective anticancer agent in nasopharyngeal carcinoma (NPG). Further exploration of the mechanism of action could yield novel breakthroughs in anticancer drug discovery. | Liu and Luo, 2012 |
| BA has anti-inflammatory and antioxidant properties that protect the lung against the deleterious effects of lipopolysaccharide. | Nader and Baraka, 2012 |
| BA significantly inhibits fibrosis by modulating the TLR4/myeloid differentiation factor 88 (MyD88)/nuclear factor (NF)-κB signaling pathway. | Wan et al., 2012 |
| BA may have anti-obesity effects by directly inhibiting pancreatic lipase. This may prevent the absorption of lipids from the small intestine. Further, BA may further accelerate fat mobilization by enhancing lipolysis in adipose tissues. | Kim et al., 2012 |

Received: January 17, 2015, accepted: January 27, 2015, published: February 02, 2015

## Acknowledgements

This study was supported by the Regional Innovation Center for Dental Science & Engineering, Chosun University, Gwangju, Korea (B0008940).

## Figures and Tables

**Table 1:**
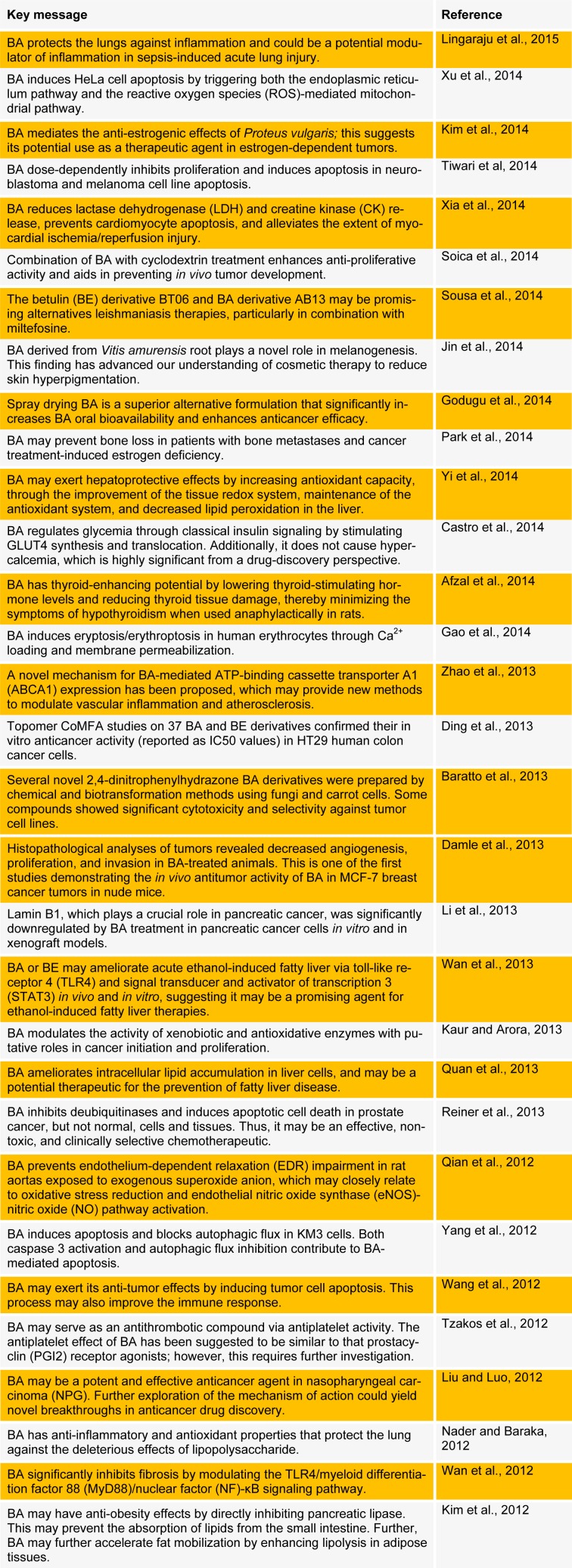
Recent studies on betulinic acid and its biological and pharmacological activities
